# An updated histology recode for the analysis of primary malignant and nonmalignant brain and other central nervous system tumors in the Surveillance, Epidemiology, and End Results Program

**DOI:** 10.1093/noajnl/vdaa175

**Published:** 2020-12-08

**Authors:** Gonçalo Forjaz, Jill S Barnholtz-Sloan, Carol Kruchko, Rebecca Siegel, Serban Negoita, Quinn T Ostrom, Lois Dickie, Jennifer Ruhl, Alison Van Dyke, Nirav Patil, Gino Cioffi, Kimberly D Miller, Kristin Waite, Angela B Mariotto

**Affiliations:** 1 Division of Cancer Control and Population Sciences, National Cancer Institute, Rockville, Maryland, USA; 2 Central Brain Tumor Registry of the United States, Hinsdale, Illinois, USA; 3 Department of Population and Quantitative Health Sciences, Case Western Reserve University School of Medicine, Cleveland, Ohio, USA; 4 Surveillance and Health Services Research, American Cancer Society, Atlanta, Georgia, USA; 5 Department of Medicine, Section of Epidemiology and Population Sciences, Dan L. Duncan Comprehensive Cancer Center, Baylor College of Medicine, Houston, Texas, USA

**Keywords:** brain tumors, classifications, incidence, trends

## Abstract

**Background:**

There are over 100 histologically distinct types of primary malignant and nonmalignant brain and other central nervous system (CNS) tumors. Our study presents recent trends in the incidence of these tumors using an updated histology recode that incorporates major diagnostic categories listed in the *2016 World Health Organization Classification of Tumours of the CNS*.

**Methods:**

We used data from the SEER-21 registries for patients of all ages diagnosed in 2000–2017. We calculated age-adjusted incidence rates and fitted a joinpoint regression to the observed data to estimate the Annual Percent Change and 95% confidence intervals over the period 2000–2017.

**Results:**

There were 315,184 new malignant (34.2%; 107,890) and nonmalignant (65.8%; 207,294) brain tumor cases during 2004–2017. Nonmalignant meningioma represented 46.5% (146,498) of all brain tumors (malignant and nonmalignant), while glioblastoma represented 50.8% (54,832) of all malignant tumors. Temporal trends were stable or declining except for nonmalignant meningioma (0.7% per year during 2004–2017). Several subtypes presented decreases in trends in the most recent period (2013–2017): diffuse/anaplastic astrocytoma (−1.3% per year, oligodendroglioma (−2.6%), pilocytic astrocytoma (−3.8%), and malignant meningioma (−5.9%).

**Conclusions:**

Declining trends observed in our study may be attributable to recent changes in diagnostic classification and the coding practices stemming from those changes. The recode used in this study enables histology reporting to reflect the changes. It also provides a first step toward the reporting of malignant and nonmalignant brain and other CNS tumors in the Surveillance, Epidemiology, and End Results (SEER) Program by clinically relevant histology groupings.

**Key Points**

- Our results show a decreasing trend in most brain tumors subtypes.
- Changes in diagnostic classification may have contributed to these trends.
- Our updated recode enables histology reporting to reflect these changes.

**Importance of the Study**

There are over 100 histologically distinct types of primary brain and other Central Nervous System (CNS) tumors, each with its own spectrum of clinical presentations, treatments, and outcomes. In the Surveillance, Epidemiology, and End Results (SEER) Program, these tumors are reported in one single category—Cancer of the Brain and Other Nervous System. We developed an updated recode that groups brain tumors into major diagnostic categories, as proposed in the *2016 World Health Organization Classification of Tumours of the CNS* (2016 CNS WHO). Our recode will allow researchers and other users of SEER data to quickly analyze brain tumors according to major histological categories that are based on those proposed in the 2016 CNS WHO, facilitating surveillance of incidence and survival for these tumors. In the future, it will also permit further stratification in some of these entities by incorporating coding changes that capture clinically meaningful brain cancer subtypes identified by molecular markers.

Primary malignant and nonmalignant brain and other central nervous system (CNS) tumors are relatively rare neoplasms, accounting for only 4% of all new primary cancers diagnosed each year in the United States.^1^ An estimated 23,890 cases of malignant brain and other CNS tumors are expected to be diagnosed in the United States in 2020 and 18,020 deaths are expected.^2^ These tumors constitute an important source of morbidity and mortality, particularly in younger patients, representing the first and second leading causes of cancer death in ages <20 and 20–39, respectively.^3^ Despite the small overall decrease in all primary brain tumor incidence rates in recent years, mortality rates have been increasing in the past few years, mainly in the elderly; an Annual Percent Change (APC) of 0.8% per year (95% confidence interval: 0.5%–1.0%) has been noted for those aged 75 years or older in the period 2000–2017.^4^

Primary nonmalignant brain and other CNS tumors include those tumors with the International Classification of Diseases for Oncology (ICD-O) behavior codes /0 (benign) and /1 (borderline) and represent more than half of all brain tumors diagnosed in the United States, with an estimated 58,860 new cases in the United States in 2020.^1^ Despite their nonmalignant nature, these tumors also constitute an important source of morbidity due to their location in the brain and CNS, as they may inhibit essential brain functions and cognition.^5^ The need for incidence data on benign and borderline primary brain tumors from patient constituents and the fact that site should guide their collection rather than behavior alone^6^ influenced Congress to pass the Benign Brain Tumor Cancer Registries Amendment Act (Public Law 107–260) in 2002.^7^ This amendment broadened the definition of reportable tumors as outlined in the original legislation (Public Law 102–515—the Cancer Registries Amendment Act—passed in 1992) to include nonmalignant brain tumors and thereby mandated the Centers for Disease Control and Prevention’s National Program of Cancer Registries to collect these tumors starting January 1, 2004. The collection of these tumors in the National Cancer Institute’s (NCI) Surveillance, Epidemiology, and End Results (SEER) Program was instituted for cases diagnosed at this same time.^8^ Following SEER’s lead, other organizations in the United States also instituted reporting of these tumors, including the North American Association of Central Cancer Registries (NAACCR) and the American College of Surgeons’ Commission on Cancer-accredited facilities.^9^ The solidarity shown in support of Public Law 107–260 enabled the Central Brain Tumor Registry of the United States (CBTRUS) to expand its former voluntary reporting of all primary brain and other CNS tumors by histology and histology groupings to the entire United States.

There are over 100 histologically distinct types of primary brain and other CNS tumors, each with its own spectrum of clinical presentations, treatments, and outcomes.^1^ In the SEER Program, these tumors are reported in one single category—Cancer of the Brain and Other Nervous System.^3^ However, aggregated data masks heterogeneity among subtypes and makes it difficult to study the risk and prognosis of distinctive brain tumor entities. Monitoring the incidence of specific histologies over time would have substantial clinical utility.

With the publication of the *2016 World Health Organization’s (WHO) Classification of Tumours of the Central Nervous System* (2016 CNS WHO),^10^ some changes within diagnostic categories occurred because molecular parameters clarified the brain tumor diagnoses or refined the prognoses.^11^ Histologic and molecular features are now embedded into the nomenclature of multiple brain tumor entities, greatly expanding the value of immunohistochemistry and fluorescent *in situ* hybridization techniques for diagnosis, prognosis, and prediction in the clinical assessment of brain tumors.^12^ These advances in diagnostic practices also enabled descriptive epidemiology studies to analyze a more precisely classified set of databases and to stratify analyses by tumor subtype more accurately.^5^

Our study aims to present recent trends in the incidence of primary malignant and nonmalignant brain and other CNS tumors using a new recode that groups these entities into major diagnostic categories proposed in the 2016 CNS WHO—mindful that this is a transition time in CNS reporting. Ideally, it would be valuable to monitor all subtypes, but because there are many distinct types, some choice needed to be made in terms of groupings to report. Of these, the more common histologies are discussed in greater detail to aid in interpreting results. Since other studies have more thoroughly analyzed the interplay between sex and race/ethnicity and brain tumor incidence,^13–16^ our study focuses primarily on the overall changes over time. This work is the first step toward reporting statistics on primary malignant and nonmalignant brain and other CNS tumors in the SEER Program by clinically relevant histology groupings.

## Methods

### Source of Data

We used data from the SEER 21 Registries Database^17^ for patients diagnosed in 2000–2017 with a primary tumor (ICD-O, 3^rd^ edition [ICD-O-3] behavior codes /0, /1 or /3)^18^ of the meninges (ICD-O-3 topography code C70._), brain (C71._), spinal cord, cranial nerves and other parts of the CNS (C72._), and pineal gland (C75.3). In contrast, CBTRUS includes sites listed in the Consensus Conference on Brain Tumor Definition.^6^ These registries cover approximately 37% of the U.S. population. Based on the standard practice used by CBTRUS in its reports, ICD-O-3 behavior codes /0 and /1 were grouped as nonmalignant and code /3 as malignant. We used only de-identified information, aggregated at the SEER 21 level, so approval by an ethics committee was not necessary.

### SEER Brain and CNS Recode

We created a condensed histology grouping—SEER Brain and CNS recode—based on the histology groupings defined in the 2016 CNS WHO (Table 1). The new recode incorporates those new histology codes implemented in this classification. The collection was initiated in the SEER Program for cases diagnosed on or after January 1, 2018.^8^ Consistent with SEER coding rules, pilocytic astrocytoma (ICD-O-3 histology code 9421) was assigned behavior code /3 (malignant), despite being coded with behavior code /1 (uncertain whether benign or malignant) in both the 2016 CNS WHO and ICD-O-3.

**Table 1.** Recode for Brain and Other Central Nervous System Tumors (SEER Brain and CNS Recode) Diagnosed in the SEER 21 Registries, 2004–2017

Category	ICD-O-3 Site	Malignant			Nonmalignant		
		ICD-O-3 Morphology	Cases	ASR	ICD-O-3 Morphology	Cases	ASR
Brain and Other Nervous System (SEER Site Recode)	C700-C729	All except 9050–9055, 9140, 9590–9992	107,890	6.5	All except 9050–9055, 9140, 9590–9992	207,294	9.9
Glioma	C700-C729	9380–9384, 9391–9460	95,198	5.7	–	–	–
Diffuse astrocytoma and anaplastic astrocytoma	C700-C729	9400/3, 9401/3, 9410/3, 9411/3, 9420/3^b^	14,363	0.9	–	–	–
Glioblastoma	C700-C729	9440/3, 9441/3, 9442/3, 9445/3^a^	54,832	3.2	–	–	–
Diffuse midline glioma, H3 K27M-mutant	C700-C729	9385/3^a^	–	–	–	–	–
Oligodendroglioma	C700-C729	9450/3, 9451/3	6,031	0.4	–	–	–
Oligoastrocytoma	C700-C729	9382/3	2,694	0.2	–	–	–
Other astrocytic tumors	C700-C729	9421/3^c^, 9424/3, 9425/3^a^	5,668	0.4	9384/1	423	0.0
Gliomas, unspecified	C700-C729	9380/3	7,472	0.5	–	–	–
Other gliomas	C700-C729	9430/3	62	0.0	9431/1,^a^ 9444/1	42	0.0
Ependymal tumors	C700-C729	9391/3, 9392/3, 9393/3, 9396/3^a^	4,076	0.3	9383/1, 9394/1	2,652	0.1
Embryonal tumors	C700-C729	8963/3, 9364/3^e^, 9470/3, 9471/3, 9472/3, 9473/3, 9474/3, 9475/3^a^, 9476/3^a^, 9477/3^a^, 9478/3^a^, 9490/3, 9500/3, 9501/3, 9502/3, 9508/3	3,581	0.2	–	–	–
Meningioma	C700-C729	9530/3, 9538/3, 9539/3	1,675	0.1	9530/0, 9531/0, 9532/0, 9533/0, 9534/0, 9537/0, 9538/1, 9539/1	146,498	7.0
Tumors of the cranial and paraspinal nerves	C700-C729	–	–	–	9540/0, 9550/0, 9560/0, 9560/1, 9571/0	33,823	1.6
Choroid plexus tumors	C700-C729	9390/3	151	0.0	9390/0, 9390/1	750	0.0
Neuronal and mixed neuronal-glial tumors	C700-C729	9505/3	191	0.0	8693/1, 9412/1, 9413/0, 9492/0, 9493/0, 9505/1, 9506/1, 9509/1^d^	3,602	0.2
Tumors of the pineal region	C753	9362/3, 9395/3^d^	427	0.0	9361/1	250	0.0
Mesenchymal, nonmeningothelial tumors	C700-C729	–	–	–	8815/0,^f^ 8815/1,^f^ 8821/1, 8825/0, 8825/1, 8830/0, 8850/0, 8861/0, 8880/0,^d^ 8890/0, 8900/0, 9120/0, 9161/1, 9180/0, 9210/0, 9220/0	5,024	0.2

ICD-O-3, International Classification of Diseases for Oncology, 3rd edition; ASR, Age-standardized rate per 100,000.

^a^New code in the 2016 CNS WHO and SEER. Effective with cases diagnosed 1/1/2018 and forward.

^b^Code 9420/3 has been deleted from the 2016 CNS WHO since this diagnosis overlaps nearly entirely with the standard diffuse astrocytoma. For that reason, it has been added to category “Diffuse astrocytoma and anaplastic astrocytoma.”

^c^The behavior for code 9421/3 is */1* in the 2016 CNS WHO but */3* in SEER and CBTRUS.

^d^New code in SEER. Effective with cases diagnosed 1/1/2018 and forward.

^e^Under “Mesenchymal, nonmeningothelial tumors” in the 2016 CNS WHO.

^f^New term. Effective with cases diagnosed 1/1/2018 and forward.

The recode includes histologies not listed under the histological groupings proposed in the 2016 CNS WHO. Two neuropathologists from CBTRUS reviewed the recode and recommended including: protoplasmic astrocytoma (ICD-O-3 morphology code 9410/3) to the “Diffuse/anaplastic astrocytoma” category; malignant rhabdoid tumor (8963/3), peripheral neuroectodermal tumor (9364/3), medullomyoblastoma (9472/3), and teratoid medulloepithelioma, NOS (9502/3) to the “Embryonal tumors” category; and meningeal sarcomatosis (9539/3) to the “Meningiomas” category. A category for unspecified types of gliomas (“Gliomas, unspecified,” 9380/3) was also included.

The new recode will be made available in NCI’s SEER*Stat and updates SEER’s “Histology recode – Brain groupings”. “A subset of the recode will also be made available in NCI’s SEER*Explorer (https://seer.cancer.gov/explorer/),^4^ an interactive tool for quick access to SEER cancer statistics.”

### Statistical Analysis

Using the SEER Brain and CNS Recode in NCI’s SEER*Stat software version 8.3.6.,^19^ we calculated incidence rates in each diagnosis year for selected histological categories, age-adjusted to the 2000 U.S. standard population and expressed per 100,000 person-years. We stratified cancer patients by age group (0–14, 15–39, 40–64, and 65+ years), sex, and race/ethnicity [non-Hispanic White (NHW), non-Hispanic Black (NHB), non-Hispanic American Indian/Alaska Natives (NHAIAN), non-Hispanic Asian or Pacific Islanders (NHAPI), and Hispanic]. We categorized age groups according to those used by CBTRUS in its Annual Statistical Report,^1^ except that we divided older adults into two categories (40–64 years and 65+ years). Standard errors and 95% confidence intervals for rates were calculated using the Tiwari method.^20^ The population estimates used as the denominators to calculate incidence rates were a modification of the intercensal and Vintage 2014.^21^

We used NCI’s Joinpoint Regression Program version 4.8.0.1 to estimate temporal trends in age-adjusted incidence rates.^22^ Due to sparse data, we excluded NHAIAN and NHAPI from this analysis. The resulting trends were described by the Annual Percent Change (APC). In describing trends, the terms “increase” or “decrease” were used when the slope of the trend was statistically significant^23^; if not, terms such as “stable,” “nonsignificant increase,” and “nonsignificant decrease” were used. We also estimated the Average Annual Percent Chance (AAPC) in the most recent period. This metric is a weighted average of the APC over a fixed interval (in our study, 2013–2017) using the whole period of the underlying joinpoint model (in our study, 2000–2017 for malignant tumors and 2004–2017 for nonmalignant tumors).^24^ Although reporting an APC for each joinpoint segment provides a complete characterization of the incidence trend over time, comparing AAPCs of equal lengths from all tumor subtypes would provide a more meaningful comparison. All hypothesis tests were two-sided, and *P* values <.05 were considered statistically significant.

## Results

Except when showing trends, we present results for the period 2004–2017 instead of 2000–2017 since figures for nonmalignant tumors are available only from 2004 onward. Overall, 315,184 new cases of brain and other CNS tumors were diagnosed in the SEER 21 registries catchment area in 2004–2017, of which 107,890 (34.2%) were malignant and 207,294 (65.8%) nonmalignant (Table 1). There were also 677 tumors of the pineal gland, of which 427 (63.1%) were malignant and 250 (36.9%) nonmalignant. Glioblastoma represented 50.8% (54,832) of all malignant tumors. This tumor is part of a broader category (Glioma), which accounted for almost 90% of all malignant brain and other CNS tumors. The incidence rate for glioblastoma was highest in males (4.1 per 100,000), persons aged 65+ years (13.4 per 100,000), and NHW (3.8 per 100,000) (Supplementary Table 1). Nonmalignant meningioma represented 46.5% (146,498) of all brain tumors combined (malignant and nonmalignant). The incidence rate for nonmalignant meningioma was highest in females (9.4 per 100,000), persons aged 65+ years (37.6 per 100,000), and NHB (10.1 per 100,000) (Supplementary Table 1).

**Figure 1.**
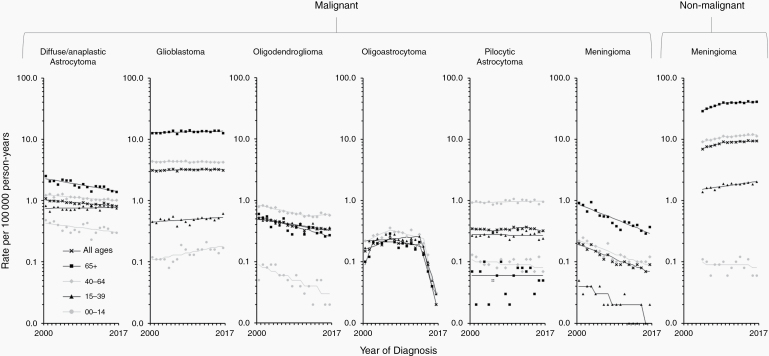
Trends in age-standardized incidence rates for selected malignant (2000–2017) and nonmalignant (2004–2017) brain and other CNS tumors in the SEER 21 registries, by age group—Both sexes. Symbols represent observed data; lines represent modeled data. The rates are plotted on the log scale.

**Figure 2.**
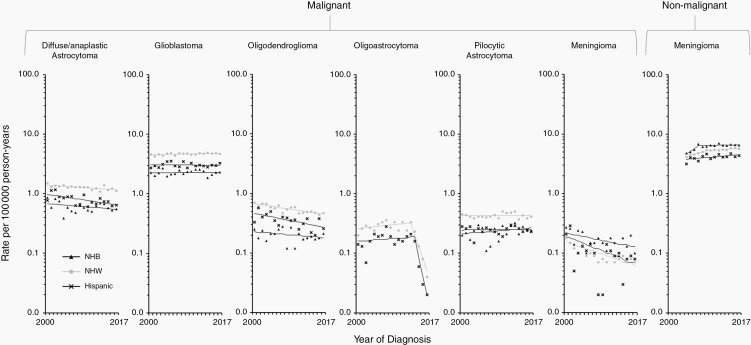
Trends in age-standardized incidence rates for selected malignant (2000–2017) and nonmalignant (2004–2017) brain and other CNS tumors in the SEER 21 registries, by race/ethnicity—Men. Symbols represent observed data; lines represent modeled data. The rates are plotted on the log scale. NHW, non-Hispanic white; NHB, non-Hispanic Black.

**Figure 3.**
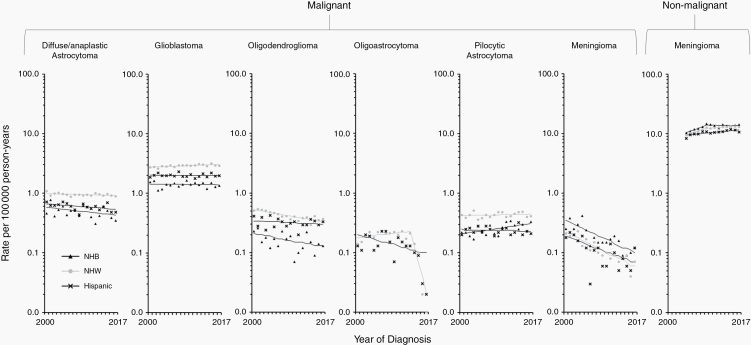
Trends in age-standardized incidence rates for selected malignant (2000–2017) and nonmalignant (2004–2017) brain and other CNS tumors in the SEER 21 registries, by race/ethnicity—Women. Symbols represent observed data; lines represent modeled data. The rates are plotted on the log scale. NHW, non-Hispanic white; NHB, non-Hispanic Black.

Trends in age-adjusted incidence rates in broader age groups as well as by sex and race/ethnicity are presented for selected malignant categories (Figures 1–3 and Supplementary Tables 2–4): diffuse/anaplastic astrocytoma, glioblastoma, oligodendroglioma (including anaplastic oligodendroglioma), oligoastrocytoma, other astrocytic tumors, and meningioma. Collectively, these accounted for almost 80% of all malignant brain and other CNS tumors diagnosed in the SEER 21 registries areas in 2004–2017 (Table 1). Since more than 90% of patients diagnosed with other astrocytic tumors corresponded to patients with a diagnosis of pilocytic astrocytoma (ICD-O-3 morphology code 9421/3), we decided to present trends for this category as representative of other astrocytic tumors. We also present trends for nonmalignant meningioma, which accounted for 70.6% of all primary nonmalignant tumors of the brain and other CNS (Table 1).

An overall decreasing trend was observed for diffuse/anaplastic astrocytoma in the most recent period, with an AAPC of −1.3% per year (95% Confidence Interval: −1.6% to −0.9%) during 2013–2017 (Figure 1 and Supplementary Table 2). A decrease was observed across all age groups except persons aged 15–39 years, among whom rates were stable. The APCs in the age-adjusted rates ranged from −2.8% per year (−3.5% to −2.0%) in those aged 65 years or older to −1.3% per year (−1.7% to −0.9%) in those aged 40–64 years. Rates increased with advancing age, being about six-fold higher in those aged 65 years or older than in those aged 0–14 years (Supplementary Table 1).

Overall incidence rates for glioblastoma have been stable in the most recent period (Figure 1 and Supplementary Table 2). In contrast to the overall trend, an increased incidence was observed from 2000–2017 among those aged 0–14 years and 15–39 years, with APCs of 3.1% per year (1.3%–5.0%) and 1.0% per year (0.1%–2.0%), respectively. Rates increased with advancing age, being about 3- and 27-fold higher in those aged 65 years or older than in those aged 40–64 years and 0–39 years, respectively (Supplementary Table 1).

An overall decreasing trend was observed for oligodendroglioma in 2013–2017, with an AAPC of −2.6% per year (−3.2% to −2.1%) (Figure 1 and Supplementary Table 2). A decrease was observed across all age groups, ranging from −6.3% per year (−9.1% to −3.5%) in those aged 0–14 years to −2.2% per year (−3.1% to −1.4%) in those aged 15–39 years. Rates peaked in those between the age 40 and 64 years (Supplementary Table 1).

An overall decreasing trend in incidence was observed for oligoastrocytoma during 2013–2017, with an AAPC of −42.4% per year (−52.2% to −30.5%) (Figure 1 and Supplementary Table 2). A decrease was observed across all age groups starting in 2013–2014. This decrease ranged from -52.6% per year (−67.4% to −31.2%) in those aged 40–64 years to -38.9% per year (−52.4% to −21.5%) in those aged 65 years or older. Trends for those aged 0–14 years could not be estimated due to low counts. Rates did not vary substantially across age groups (Supplementary Table 1).

An overall decreasing trend was observed for pilocytic astrocytoma in 2013–2017, with an AAPC of −3.8% per year (−6.9% to −0.6%) (Figure 1 and Supplementary Table 2). Rates decreased with advancing age, being about 10-fold higher in those aged 0–14 years than in those aged 65 years or older (Supplementary Table 1).

An overall decreasing trend was observed for malignant meningioma in 2013–2017, with an AAPC of −5.9% per year (−7.1% to −4.8%) (Figure 1 and Supplementary Table 2). A decrease was observed across all age groups. This decrease ranged from −6.9% per year (−9.1% to −4.5%) in those aged 15–39 years to −5.5% per year (−7.1% to −3.8%) in those aged 40–64 years. Trends for those aged 0–14 years could not be estimated due to low counts. Rates peaked in those aged 65 years or older (Supplementary Table 1).

An overall increasing trend was observed for nonmalignant meningioma in 2013–2017, with an AAPC of 0.7% per year (0.2%–1.2%) (Figure 1 and Supplementary Table 2). An increase was observed across all age groups except 0–14 years, with the largest increase occurring in those aged 65 years or older during 2004–2009 (5.8% per year [4.4%–7.2%]), after which it stabilized. Rates increased with advancing age, being about 21-fold higher in those aged 65 or older than in those aged 15–39 years (Supplementary Table 1). Rates in females were 2.3 times those reported for males (Supplementary Table 1).

## Discussion

In this study, we used an updated histology recode—SEER Brain and CNS Recode—to report recent trends in the incidence of brain and other CNS tumors in the SEER 21 registries catchment area during 2000–2017. Overall, our results show a decreasing trend in most histological groups. In the most recent period (2013–2017), a decrease was observed for diffuse/anaplastic astrocytoma (−1.3%/year), oligodendroglioma (−2.6%/year), oligoastrocytoma (−42.4%/year), pilocytic astrocytoma (−3.7%/year), and malignant meningioma (−5.9%/year). Some studies have shown downward trends similar to those observed in our study,^25,26^ but others have reported increasing trends.^27,28^ Improved reporting and increased use of diagnostic imaging are two of the factors suspected to account for the increases observed in the latter.^29^ Despite our analysis being focused on overall incidence trends, we also show that NHW had the highest incidence across most histological groups, consistent with other published studies.^15,16,30^ Greater access to health care, higher socioeconomic status, and variation in inherited genetic risk are some of the potential factors that may explain part of the incidence difference between NHW and other races/ethnicities.

In the previous revision of the WHO Classification (2007 CNS WHO),^31^ the authors proposed reclassifying high-grade tumors with oligodendroglial/astrocytic characteristics as glioblastoma with oligodendroglioma components. It was also postulated that the availability and implementation of molecular technologies to test for genetic abnormalities (ie, fluorescent in situ hybridization for detecting 1p/19q co-deletion) improved diagnostic accuracy. Together, these changes could have contributed to a decrease in astrocytoma and oligodendroglioma in age groups 0–14 and 15–39 years (Figure 1 and Supplementary Table 2). These were the same age groups in which an increasing trend was noted for glioblastoma, indicating that some shifts between categories may have occurred. The increasing use of molecular characterization in the diagnostic workup may also have impacted the young and the old differently. Younger patients with glioblastoma have better outcomes when *IDH1*/2 mutations and *MGMT* promoter methylation are present, so an accurate diagnosis is critical for these patients.^32^

However, the downward trend observed for astrocytoma and oligodendroglioma in older age groups (40–64 years and 65+ years) has not been paralleled by an increase in glioblastoma across the same age groups. On the contrary, incidence trends in glioblastoma have been relatively stable in those aged 40 years or older (Figure 1 and Supplementary Table 2), in whom more than 90% of cases are diagnosed (Supplementary Table 1). Future studies should explore factors contributing to the decreasing trend observed in most of the glioma subtypes. One promising area to investigate is the inverse association between allergies, including asthma and eczema, and risk of glioma, as found in numerous studies.^33^ This association is the most recent in brain tumor epidemiology to reach scientific consensus.^34^

Two studies, one from Sweden and the other from England, have reported a sustained rise in glioblastoma during 1998–2013 and 1995–2015, respectively.^27,28^ Our study does not report a similar rise in glioblastoma overall nor in those age groups in which it is more prevalent (40 years or older). This inconsistency may be the result of demographic differences between these more homogenous populations and the United States, exacerbated by the overrepresentation of minority populations in SEER registries. One of the most important features of the SEER database is that it covers diverse populations within the United States.^35^ Even though rates in NHW were twice those reported for NHB and 1.5 times those reported for Hispanic (Supplementary Table 1), proportionally more NHB (34.7% of all Blacks in the United States) and Hispanic (46.7% of all Hispanics in the United States) than NHW (33.6% of all Whites in the United States) are covered in the SEER 21 areas. When stratified by race/ethnicity, our results show that trends for glioblastoma were stable for NHB and Hispanic in both males and females, while increasing slightly in NHW males (0.4% [0.0%–0.7%] per year) and females (0.6% [0.3%–0.9%] per year) (Figures 2–3 and Supplementary Tables 3–4). Incidence rates encompassing all races/ethnicities may thus be inappropriate when comparing with other populations, namely those in which Blacks and Hispanics make up a smaller proportion of the population, such as in Sweden or England. When comparing to jurisdictions outside of the United States, we recommend some caution in interpreting results when stratification by race/ethnicity is not possible.

Trend figures for oligoastrocytoma are a good example of how changes in classification due to improving diagnostic practices impact incidence trends over time. The 2007 CNS WHO had already proposed deemphasizing the mixed glioma phenotype (also known as oligoastrocytoma),^31^ but in 2014 Sahm and colleagues published a study where they recommended refraining from diagnosing oligoastrocytoma and instead classifying it either as oligodendroglioma or astrocytoma.^36^ They provided evidence that the use of molecular markers segregates tumors that appear histologically to be oligoastrocytoma into two groups, matching the genetic characteristics of oligodendroglioma or astrocytoma. Our results show a steep decrease in incidence across all age groups starting in 2013–2014 (Figure 1), consistent with changes in diagnostic criteria. These changes led the 2016 CNS WHO to strongly discourage the diagnosis of oligoastrocytoma (or any of its synonyms) as a valid glioma histologic subtype.^11^ Changes have not been reflected in statistically significantly increased rates of astrocytoma and oligodendroglioma, but this may simply be due to differences in incidence rates’ scale (eg, overall rates are 5.3 and 2.3 times higher, respectively, than those for oligoastrocytoma).

Nonmalignant meningioma incidence may have increased simply due to reporting learning curves associated with the implementation of Public Law 107-260,^37^ including an improved collection of radiographically diagnosed cases.^1^ However, changes in classification may also explain part of the increase, particularly the adoption of the 2000 and 2007 guidelines of the WHO Classification of Central Nervous System Tumors, which downgraded cases of meningioma with brain invasion but without anaplasia from grade III (malignant) to grade II or I (nonmalignant).^38^ Part of the decline in malignant meningioma incidence may have resulted from this shift from malignant to nonmalignant. However, the decline in malignant meningioma started before the Benign Brain Tumor Cancer Registries Amendment Act came into effect (2004). Future research is warranted to elucidate the causes of the decreasing trends noted for malignant meningioma, particularly in those 65 years of age or older (Figure 1 and Supplementary Table 2). Nonmalignant meningioma is also one of the few brain tumor subtypes that is more common in females than males. Despite some studies having suggested a role for hormonally mediated risk factors,^39,40^ specific mechanisms accounting for this sex difference remain elusive.

Our study has several limitations. First, the updated recode does not include stratification by molecular markers, as these started to be collected only for cases diagnosed on or after January 1, 2018. However, an update including these genetic markers will be made available with the 2018 SEER data release in 2021, permitting more granular analysis. Second, our study’s histology grouping scheme differs slightly from that used currently by CBTRUS, which leverages data from 100% of the United States (50 state cancer registries and the District of Columbia).^3^ Therefore, direct one-to-one comparison of statistics generated from these two sources is not possible. We based ours on the 2016 CNS WHO since this classification reflects changes that occurred more recently—due to the incorporation of molecular findings into brain tumor diagnoses. While the histologies with molecular markers are not currently available to report, these histologies have been incorporated into our grouping scheme in consultation with CBTRUS, which will also start reporting results by molecular markers in 2021. Third, despite the use of SEER 21 registries data, which cover around 36.7% of the U.S. population, the incidence trends presented in our study may not be representative of the country’s population subgroups. Finally, our recode excludes some of the less frequent histological groupings mentioned in the 2016 CNS WHO. In the malignant categories, these tumors represented around 6.7% of all primary malignant brain and other CNS tumors diagnosed in 2004–2017 (data not shown), while in the nonmalignant categories, they represented around 7.0% of all primary nonmalignant brain and other CNS tumors diagnosed in 2004–2017 (data not shown). The rationale for excluding these categories is that they are very rare entities, making the analyses of trends over time challenging to interpret.

In conclusion, the updated recode will allow researchers and other users of SEER data to quickly analyze brain tumors according to major histological categories that are based on those proposed in the 2016 CNS WHO, facilitating surveillance of incidence and survival for these tumors, by sex, age group, and race/ethnicity. It will also permit further stratification in some of these entities by incorporating coding changes introduced in the SEER Program for cases diagnosed in or after 2018. These changes pertain specifically to the inclusion of a new Site-Specific Data Item—*Brain Molecular Markers*—in the NAACCR’s metafile,^41^ as proposed by CBTRUS.^42^ The new item captures clinically meaningful brain cancer subtypes identified by molecular markers that are not distinguishable by ICD-O-3 codes,^41^ and it will aid and inform future studies of population-based disease trends. As for the trends observed in our study, they are more likely attributable to a combination of changes in coding practices and diagnoses than to actual changes in the risk of brain and other CNS tumors. That was certainly the case with oligoastrocytoma, and probably with nonmalignant meningioma.

## Supplementary Material

vdaa175_suppl_Supplementary_Table_1

vdaa175_suppl_Supplementary_Table_2

vdaa175_suppl_Supplementary_Table_3

vdaa175_suppl_Supplementary_Table_4

## Notes

**Authorship Statement.** Analysis and interpretation of the data—all authors. Writing of the manuscript at the draft and any revision stages—all authors. Reading and approval of the final version—all authors.
