# Supplementary material for: An updated histology recode for the analysis of primary malignant and nonmalignant brain and other central nervous system tumors in the Surveillance, Epidemiology, and End Results Program
Source: Neurooncol Adv. 2020 Dec 8;3(1):vdaa175. doi: 10.1093/noajnl/vdaa175 (PMC7813198; doi:10.1093/noajnl/vdaa175)
Supplement: vdaa175_suppl_Supplementary_Table_1 [file vdaa175_suppl_supplementary_table_1.docx]

| **Supplementary Table 1.** Demographic characteristics of patients diagnosed with selected subtypes of a tumor of the brain and other central nervous system in the SEER 21 registries, 2004–2017 | | | | | | | | | | | | | | | | | | | | |
| --- | --- | --- | --- | --- | --- | --- | --- | --- | --- | --- | --- | --- | --- | --- | --- | --- | --- | --- | --- | --- |
|  | **Diffuse Astrocytoma and Anaplastic Astrocytoma** | |  | **Glioblastoma** | |  | **Oligodendroglioma** | |  | **Oligoastrocytoma** | |  | **Other Astrocytic Tumors** | |  | **Malignant Meningioma** | |  | **Non-malignant Meningioma** | |
|  | **New Cases (%)** | **Rate (95% CI)** |  | **New Cases (%)** | **Rate (95% CI)** |  | **New Cases (%)** | **Rate (95% CI)** |  | **New Cases (%)** | **Rate (95% CI)** |  | **New Cases (%)** | **Rate (95% CI)** |  | **New Cases (%)** | **Rate (95% CI)** |  | **New Cases (%)** | **Rate (95% CI)** |
| **Sex** |  |  |  |  |  |  |  |  |  |  |  |  |  |  |  |  |  |  |  |  |
| Male | 7,990 (55.6) | 1.0 (1.0 to 1.0) |  | 31,734 (57.9) | 4.1 (4.0 to 4.1) |  | 3,338 (55.3) | 0.4 (0.4 to 0.4) |  | 1,564 (58.1) | 0.2 (0.2 to 0.2) |  | 2,888 (58.1) | 0.4 (0.4 to 0.4) |  | 700 (41.8) | 0.1 (0.1 to 0.1) |  | 38,158 (26.0) | 4.1 (4.1 to 4.2) |
| Female | 6,373 (44.4) | 0.8 (0.7 to 0.8) |  | 23,098 (42.1) | 2.5 (2.5 to 2.5) |  | 2,693 (44.7) | 0.3 (0.3 to 0.3) |  | 1,130 (41.9) | 0.1 (0.1 to 0.1) |  | 2,780 (41.9) | 0.4 (0.4 to 0.4) |  | 975 (58.2) | 0.1 (0.1 to 0.1) |  | 108,340 (74.0) | 9.4 (9.4 to 9.5) |
|  |  |  |  |  |  |  |  |  |  |  |  |  |  |  |  |  |  |  |  |  |
| **Age (years)** |  |  |  |  |  |  |  |  |  |  |  |  |  |  |  |  |  |  |  |  |
| 00–14 | 1,052 (7.3) | 0.3 (0.3 to 0.4) |  | 464 (0.8) | 0.1 (0.1 to 0.2) |  | 138 (2.3) | 0.0 (0.0 to 0.1) |  | 76 (2.8) | 0.0 (0.0 to 0.0) |  | 3,154 (55.6) | 1.0 (1.0 to 1.0) |  | 15 (0.9) | 0.0 (0.0 to 0.0) |  | 274 (0.2) | 0.1 (0.1 to 0.1) |
| 15–39 | 4,131 (28.8) | 0.8 (0.7 to 0.8) |  | 2,638 (4.8) | 0.5 (0.5 to 0.5) |  | 2,059 (34.1) | 0.4 (0.4 to 0.4) |  | 1,085 (40.3) | 0.2 (0.2 to 0.2) |  | 1,816 (32.0) | 0.3 (0.3 to 0.3) |  | 107 (6.4) | 0.0 (0.0 to 0.0) |  | 9,250 (6.3) | 1.8 (1.7 to 1.8) |
| 40–64 | 5,711 (39.8) | 1.1 (1.0 to 1.1) |  | 24,596 (44.9) | 4.3 (4.3 to 4.4) |  | 3,129 (51.9) | 0.6 (0.6 to 0.6) |  | 1,210 (44.9) | 0.2 (0.2 to 0.3) |  | 563 (9.9) | 0.1 (0.1 to 0.1) |  | 674 (40.2) | 0.1 (0.1 to 0.1) |  | 59,975 (40.9) | 10.9 (10.8 to 11.0) |
| 65+ | 3,469 (24.2) | 1.7 (1.7 to 1.8) |  | 27,134 (49.5) | 13.4 (13.2 to 13.5) |  | 705 (11.7) | 0.3 (0.3 to 0.4) |  | 323 (12) | 0.2 (0.1 to 0.2) |  | 135 (2.4) | 0.1 (0.1 to 0.1) |  | 879 (52.5) | 0.4 (0.4 to 0.5) |  | 76,999 (52.6) | 37.6 (37.4 to 37.9) |
|  |  |  |  |  |  |  |  |  |  |  |  |  |  |  |  |  |  |  |  |  |
| **Race^a^** |  |  |  |  |  |  |  |  |  |  |  |  |  |  |  |  |  |  |  |  |
| NHW | 10,673 (74.6) | 1.1 (1.1 to 1.1) |  | 44,008 (80.4) | 3.8 (3.7 to 3.8) |  | 4,332 (72.3) | 0.5 (0.4 to 0.5) |  | 1,942 (72.5) | 0.2 (0.2 to 0.2) |  | 3,604 (64.5) | 0.5 (0.4 to 0.5) |  | 1,071 (64.2) | 0.1 (0.1 to 0.1) |  | 101,885 (70.1) | 8.8 (8.8 to 8.9) |
| NHB | 943 (6.6) | 0.5 (0.5 to 0.5) |  | 3,072 (5.6) | 1.8 (1.7 to 1.8) |  | 313 (5.2) | 0.2 (0.1 to 0.2) |  | 139 (5.2) | 0.1 (0.1 to 0.1) |  | 562 (10.1) | 0.3 (0.3 to 0.3) |  | 256 (15.3) | 0.2 (0.1 to 0.2) |  | 16,693 (11.5) | 10.1 (10.0 to 10.3) |
| NHAIAN | 73 (0.5) | 0.7 (0.5 to 0.8) |  | 159 (0.3) | 1.5 (1.3 to 1.8) |  | 40 (0.7) | 0.4 (0.3 to 0.5) |  | 20 (0.7) | 0.2 (0.1 to 0.3) |  | 43 (0.8) | 0.4 (0.3 to 0.5) |  | 7 (0.4) | 0.1 (0.0 to 0.1) |  | 724 (0.5) | 7.4 (6.9 to 8.0) |
| NHAPI | 769 (5.4) | 0.5 (0.5 to 0.6) |  | 2,251 (4.1) | 1.6 (1.5 to 1.6) |  | 385 (6.4) | 0.3 (0.2 to 0.3) |  | 176 (6.6) | 0.1 (0.1 to 0.1) |  | 300 (5.4) | 0.2 (0.2 to 0.2) |  | 152 (9.1) | 0.1 (0.1 to 0.1) |  | 10,299 (7.1) | 7.4 (7.3 to 7.5) |
| Hispanic | 1,841 (12.9) | 0.7 (0.6 to 0.7) |  | 5,236 (9.6) | 2.5 (2.4 to 2.6) |  | 923 (15.4) | 0.3 (0.3 to 0.3) |  | 402 (15) | 0.1 (0.1 to 0.1) |  | 1,075 (19.3) | 0.3 (0.3 to 0.3) |  | 183 (11) | 0.1 (0.1 to 0.1) |  | 15,790 (10.9) | 7.6 (7.5 to 7.7) |
| Rates are per 100,000 and age-adjusted to the 2000 U.S. Standard Population CI, Confidence interval; NHW, non-Hispanic White; NHB, non-Hispanic Black; NHAIAN, non-Hispanic American Indian/Alaska Native; NHAPI, non-Hispanic Asian or Pacific Islander ^a^Unknown race excluded | | | | | | | | | | | | | | | | | | | | |
