# Supplementary material for: An updated histology recode for the analysis of primary malignant and nonmalignant brain and other central nervous system tumors in the Surveillance, Epidemiology, and End Results Program
Source: Neurooncol Adv. 2020 Dec 8;3(1):vdaa175. doi: 10.1093/noajnl/vdaa175 (PMC7813198; doi:10.1093/noajnl/vdaa175)
Supplement: vdaa175_suppl_Supplementary_Table_2 [file vdaa175_suppl_supplementary_table_2.docx]

| **Supplementary Table 2.** Trends in incidence for selected malignant (2000–2017) and non-malignant (2004–2017) brain and other CNS tumors in the SEER 21 registries using the Joinpoint Regression Program, by age group | | | | | | | | | | |
| --- | --- | --- | --- | --- | --- | --- | --- | --- | --- | --- |
| **Category** | **JP Trend 1** | |  | **JP Trend 2** | |  | **JP Trend 3** | |  | **AAPC** |
|  | **Years** | **APC** |  | **Years** | **APC** |  | **Years** | **APC** |  | **2013–2017** |
| Diffuse astrocytoma and anaplastic astrocytoma |  |  |  |  |  |  |  |  |  |  |
| 00–14 | 2000–2017 | -2.0* |  | – | – |  | – | – |  | -2.0* |
| 15–39 | 2000–2017 | 0.3 |  | – | – |  | – | – |  | 0.3 |
| 40–64 | 2000–2017 | -1.3* |  | – | – |  | – | – |  | -1.3* |
| 65+ | 2000–2017 | -2.8* |  | – | – |  | – | – |  | -2.8* |
| All ages | 2000–2017 | -1.3* |  | – | – |  | – | – |  | -1.3* |
| Glioblastoma |  |  |  |  |  |  |  |  |  |  |
| 00–14 | 2000–2017 | 3.1* |  | – | – |  | – | – |  | 3.1* |
| 15–39 | 2000–2017 | 1.0* |  | – | – |  | – | – |  | 1.0* |
| 40–64 | 2000–2017 | -0.1 |  | – | – |  | – | – |  | -0.1 |
| 65+ | 2000–2017 | 0.3 |  | – | – |  | – | – |  | 0.3 |
| All ages | 2000–2017 | 0.2 |  | – | – |  | – | – |  | 0.2 |
| Oligodendroglioma |  |  |  |  |  |  |  |  |  |  |
| 00–14 | 2000–2017 | -6.3* |  | – | – |  | – | – |  | -6.3* |
| 15–39 | 2000–2017 | -2.2* |  | – | – |  | – | – |  | -2.2* |
| 40–64 | 2000–2012 | -3.5* |  | 2012–2017 | 1.8 |  | – | – |  | -2.4* |
| 65+ | 2000–2017 | -4.2* |  | – | – |  | – | – |  | -4.2* |
| All ages | 2000–2017 | -2.6* |  | – | – |  | – | – |  | -2.6* |
| Oligoastrocytoma |  |  |  |  |  |  |  |  |  |  |
| 00–14 | – | – |  | – | – |  | – | – |  | – |
| 15–39 | 2000–2013 | 1.4 |  | 2013–2017 | -40.1* |  | – | – |  | -40.1* |
| 40–64 | 2000–2006 | 7.8* |  | 2006–2014 | -3.9 |  | 2014–2017 | -52.6* |  | -43.5* |
| 65+ | 2000–2002 | 51.9 |  | 2002–2013 | -1.2 |  | 2013–2017 | -38.9* |  | -38.9* |
| All ages | 2000–2006 | 7.3* |  | 2006–2014 | -4.0* |  | 2014–2017 | -51.4* |  | -42.4* |
| Pilocytic astrocytoma^a^ |  |  |  |  |  |  |  |  |  |  |
| 00–14 | 2000–2017 | 0.3 |  | – | – |  | – | – |  | 0.3 |
| 15–39 | 2000–2017 | -0.2 |  | – | – |  | – | – |  | -0.2 |
| 40–64 | 2000–2017 | -1.5 |  | – | – |  | – | – |  | -1.5 |
| 65+ | 2000–2017 | -0.2 |  | – | – |  | – | – |  | -0.2 |
| All ages | 2000–2005 | -1.6 |  | 2005–2013 | 1.6* |  | 2013–2017 | -3.8* |  | -3.8* |
| Meningioma (malignant) |  |  |  |  |  |  |  |  |  |  |
| 00–14 | – | – |  | – | – |  | – | – |  | – |
| 15–39 | 2000–2017 | -6.9* |  | – | – |  | – | – |  | -6.9* |
| 40–64 | 2000–2017 | -5.5* |  | – | – |  | – | – |  | -5.5* |
| 65+ | 2000–2017 | -6.2* |  | – | – |  | – | – |  | -6.2* |
| All ages | 2000–2017 | -5.9* |  | – | – |  | – | – |  | -5.9* |
| Meningioma (non-malignant) |  |  |  |  |  |  |  |  |  |  |
| 00–14 | 2004–2017 | -1.0 |  | – | – |  | – | – |  | -1.0 |
| 15–39 | 2004–2017 | 2.2* |  | – | – |  | – | – |  | 2.2* |
| 40–64 | 2004–2009 | 3.7* |  | 2009–2017 | 0.9* |  | – | – |  | 0.9* |
| 65+ | 2004–2009 | 5.8* |  | 2009–2017 | 0.5 |  | – | – |  | 0.5 |
| All ages | 2004–2009 | 4.9* |  | 2009–2017 | 0.7* |  | – | – |  | 0.7* |
| ^a^Pilocytic astrocytoma is part of "Other astrocytic tumors" in the SEER Brain and CNS Recode *The APC/AAPC is significantly different from zero (p<0.05) JP, Joinpoint; APC, Annual percent change; AAPC, Average annual percent change | | | | | | | | | | |
