# Supplementary material for: An updated histology recode for the analysis of primary malignant and nonmalignant brain and other central nervous system tumors in the Surveillance, Epidemiology, and End Results Program
Source: Neurooncol Adv. 2020 Dec 8;3(1):vdaa175. doi: 10.1093/noajnl/vdaa175 (PMC7813198; doi:10.1093/noajnl/vdaa175)
Supplement: vdaa175_suppl_Supplementary_Table_4 [file vdaa175_suppl_supplementary_table_4.docx]

| **Supplementary Table 4.** Trends in incidence for selected malignant (2000–2017) and non-malignant (2004–2017) brain and other CNS tumors in the SEER 21 registries using the Joinpoint Regression Program, by race/ethnicity — Women | | | | | | | |
| --- | --- | --- | --- | --- | --- | --- | --- |
| **Category** | **JP Trend 1** | |  | **JP Trend 2** | |  | **AAPC** |
|  | **Years** | **APC** |  | **Years** | **APC** |  | **2013–2017** |
| Diffuse astrocytoma and anaplastic astrocytoma |  |  |  |  |  |  |  |
| Non-Hispanic White | 2000–2017 | -0.5 |  | – | – |  | -0.5 |
| Non-Hispanic Black | 2000–2017 | -1.7 |  | – | – |  | -1.7 |
| Hispanic | 2000–2017 | -1.2* |  | – | – |  | -1.2* |
| Glioblastoma |  |  |  |  |  |  |  |
| Non-Hispanic White | 2000–2017 | 0.6* |  | – | – |  | 0.6* |
| Non-Hispanic Black | 2000–2017 | -0.2 |  | – | – |  | -0.2 |
| Hispanic | 2000–2017 | 0.0 |  | – | – |  | 0.0 |
| Oligodendroglioma |  |  |  |  |  |  |  |
| Non-Hispanic White | 2000–2017 | -2.7* |  | – | – |  | -2.7* |
| Non-Hispanic Black | 2000–2017 | -3.0* |  | – | – |  | -3.0* |
| Hispanic | 2000–2017 | -0.7 |  | – | – |  | -0.7 |
| Oligoastrocytoma |  |  |  |  |  |  |  |
| Non-Hispanic White | 2000–2013 | 2.0 |  | 2013–2017 | -43.0* |  | -11.1* |
| Non-Hispanic Black | – | – |  | – | – |  | – |
| Hispanic | 2000–2017 | -4.2 |  | – | – |  | -4.2 |
| Pilocytic astrocytoma^a^ |  |  |  |  |  |  |  |
| Non-Hispanic White | 2000–2017 | 0.3 |  | – | – |  | 0.3 |
| Non-Hispanic Black | 2000–2017 | 2.5* |  | – | – |  | 2.5* |
| Hispanic | 2000–2017 | -0.3 |  | – | – |  | -0.3 |
| Meningioma (malignant) |  |  |  |  |  |  |  |
| Non-Hispanic White | 2000–2017 | -7.4* |  | – | – |  | -7.4* |
| Non-Hispanic Black | 2000–2017 | -7.1* |  | – | – |  | -7.1* |
| Hispanic | 2000–2017 | -5.9* |  | – | – |  | -5.9* |
| Meningioma (non-malignant) |  |  |  |  |  |  |  |
| Non-Hispanic White | 2004–2009 | 5.2* |  | 2009–2017 | 1.0* |  | 2.6* |
| Non-Hispanic Black | 2004–2009 | 6.0* |  | 2009–2017 | -0.1 |  | 2.2* |
| Hispanic | 2004–2017 | 1.5* |  | – | – |  | 1.5* |
| ^a^Pilocytic astrocytoma is part of "Other astrocytic tumors" in the SEER Brain and CNS Recode *The APC/AAPC is significantly different from zero (p<0.05) JP, Joinpoint; APC, Annual percent change; AAPC, Average annual percent change | | | | | | | |
